# Potential of patient-reported outcomes as nonprimary endpoints in clinical trials

**DOI:** 10.1186/1477-7525-11-83

**Published:** 2013-05-15

**Authors:** Ari Gnanasakthy, Sandra Lewis, Marci Clark, Margaret Mordin, Carla DeMuro

**Affiliations:** 1 Novartis Pharmaceuticals Corporation, One Health Plaza, East Hanover, NJ, USA; 2 Health Solutions, 200 Park Offices Drive, Research Triangle Park, NC 27709, USA; 3 RTI Health Solutions, 3005 Boardwalk St., Suite 105, Ann Arbor, MI 48108, USA

**Keywords:** Clinical trial endpoints, Patient-reported outcomes, Medical labeling

## Abstract

**Background:**

The purpose of this research was to fully explore the impact of endpoint type (primary vs. nonprimary) on decisions related to patient-reported outcome (PRO) labeling claims supported by PRO measures and to determine if nonprimary PRO endpoints are being fully optimized.

This review examines the use of PROs as both primary and nonprimary endpoints in support of demonstration of treatment benefit of new molecular entities (NMEs) and biologic license applications (BLAs) in the United States in the years 2000 to 2012.

**Methods:**

All NMEs and BLAs approved by the Food and Drug Administration (FDA) between January 2000 and June 2012 were identified using the FDA Drug Approval Reports Web page. Generic products granted tentative approvals were excluded. For all identified products, medical review sections from publicly available drug approval packages were reviewed to identify PRO endpoint status. Product labels (indication, clinical trials sections) were reviewed to determine the number and type of PRO claim.

**Results:**

A total of 308 NMEs/BLAs were identified. Of these, 70 NMEs/BLAs (23%) were granted PRO claims. The majority of product claims were for disease- or condition-specific signs and symptoms. Of the 70 products with PRO claims, a PRO was a primary endpoint for the vast majority (57 [81%]). A total of 19 of the 70 products were granted a PRO claim based on a nonprimary endpoint. While nonprimary endpoints were used most often to support claims of improved signs or symptoms, nonprimary endpoints were much more likely to support claims of higher order impacts.

**Conclusions:**

Successful PRO labeling claims are typically based on primary endpoints assessing signs and symptoms. Based on this research, studies with PROs as primary endpoints are far more likely to facilitate positive regulatory review and acceptance of PROs in support of labeling claims. Although inclusion of PROs as nonprimary endpoints in clinical trials has its challenges, recent PRO labels granted by the FDA show that they can indeed be candidates for PRO labeling claims as long as they are supported by evidence.

## Background

Patient-reported outcome (PRO) is an umbrella term used to describe data collected directly from the patient without interpretation by clinicians or others [1-3]. PRO data are collected using standardized questionnaires, diaries, or event logs that are designed to measure an explicit concept (construct), such as signs and symptoms of disease, functioning (activity limitations), health status/health-related quality of life (HRQOL), and treatment satisfaction. PROs are frequently included in registration clinical trials for multiple purposes, including support of product approvals and labeling claims of treatment benefit, support of primary endpoints, and to provide a basis for publication and communication strategies. Also, increased competition in a global market requires pharmaceutical companies to seek methods to differentiate their products from those of competitors. One way of achieving this goal is to generate value propositions that extend beyond the traditional safety and clinical efficacy messages and allow competition on something other than price alone. PRO measures provide this necessary insight into patient experience of treatment benefit.

Primary endpoints are intended to address the most important research being addressed by a trial. In the absence of objective clinical endpoints to support efficacy, PRO measures may be used as primary endpoints. PROs used as primary endpoints are typically those that directly assess drug efficacy by measuring disease-defining signs and symptoms. Examples of indications for which PROs consistently serve as primary endpoints include painful conditions, such as migraine and neuropathic pain, as well as functional gastrointestinal disorders.

PROs may also be used in clinical trials as nonprimary endpoints to provide support for primary endpoints. For example, among conditions for which there are well-accepted clinician-reported primary endpoints, such as depression and other psychiatric indications, PRO measures addressing patients’ perceptions of their symptom severity, such as the Beck Depression Inventory or the Zung Self-Rating Depression Scale, are often used as key secondary endpoints. In addition to signs and symptoms, nonprimary PRO endpoints often assess higher order constructs such as functional status and HRQOL. In many instances, PROs are included in confirmatory trials at the request of regulatory bodies, such as the FDA and the European Medicines Agency (EMA) [4] or to secure reimbursement [5].

Sponsors commonly include nonprimary PRO endpoints to demonstrate value propositions of new medicines to stakeholders other than regulatory agencies [6,7]. For example, demonstration of improvement in work productivity is important in the treatment of rheumatoid arthritis [8]. Nonprimary PRO endpoints also may be included in clinical trials to satisfy health technology assessment (HTA) requirements. For example, the National Institute for Health and Clinical Excellence (NICE) has recommended patient scores in Adult Growth Hormone Deficiency scale (QoL-AGHDA) as one of the three criteria for judging patient suitability for treatment with recombinant human growth hormone [6], and the EQ-5D is included in most confirmatory clinical trials to demonstrate the economic value propositions of new medicines. Risks of not including a PRO include inaccurate reporting or understanding of patient symptoms and symptom severity related to treatment, important prognostic information, and insight into patient decision making and adherence to therapy [9].

A recent review of PRO labels between 2006 and 2010 found that almost half (45%) of all drug approval package submissions included PROs and that a vast majority of PRO labeling claims (71%) were granted for evidence based on the primary endpoint [10]. The purpose of this research is to fully explore the impact of endpoint type (primary vs. nonprimary) on resultant labeling claims over a 12 ½ -year period supported by PRO measures and determine if nonprimary endpoints are being fully optimized. This review will further contribute to continued analyses of the role of PROs in clinical trials and provide a broader perspective, including an examination of the nonprimary endpoints pre- and postrelease of the FDA PRO guidance [3].

## Methods

The methods for the data collection component for this research are fully described in a previous publication [10] and are similar to those published by Willke and colleagues [11]. Briefly, all new molecular entities (NMEs) and biological license applications (BLAs) for new prescription drugs approved in the United States (US) from January 2000 through June 2012 were reviewed. Products were excluded if they contained substances previously marketed with a different brand name or set of indications, in a different dosage form or strength, or as a combination product of previously marketed entities.

Once products were identified, drug approval packages (DAP) and approved product labels were extracted by a single researcher and reviewed by all authors. As available, information was retrieved from the medical review, summary review, cross-discipline team leader review, and other review sections from the DAP, as well as the indication and clinical studies section of the approved product label. The DAPs were located on the FDA Web site, Drugs@FDA [12]. In most cases, the label was found on the FDA Web site under approval history for the drug at time of approval. In the event the approved label was unavailable for the specified timeframe, the current label was used. Relevant information was scrutinized for each product to determine the indication, use of PRO, and PRO claim language. Statistical analysis consisted of simple frequencies and cross-tabulations of measured characteristics. Calculations were performed using Microsoft Excel 2007.

## Results

A total of 308 NMEs/BLAs were identified between the years 2000 and 2012. Of these, 70 (23%) NMEs/BLAs were granted a total of 83 PRO claims. A PRO was included as a primary endpoint for 57 of the 70 products (81%). The majority of product claims granted were for disease- or condition-specific signs and symptoms. Fewer claims included higher order measures such as functioning, HRQOL, or patient global ratings. Table 1 depicts the products that were granted PRO labeling claims by type of claim.

**Table 1:** PRO labeling claims by type of claim (2000-2012)

**Type of claim (N = 83)**	**Products with PRO claim (N = 70)**	
	**Number of claims**	**Percentage of claims**
Signs and symptoms	59	84
Functioning	13	19
HRQOL	3	4
Patient global rating	3	4
Other	5	7

*HRQOL*, health-related quality of life; *PRO*, patient-reported outcome.

**Figure 1:**
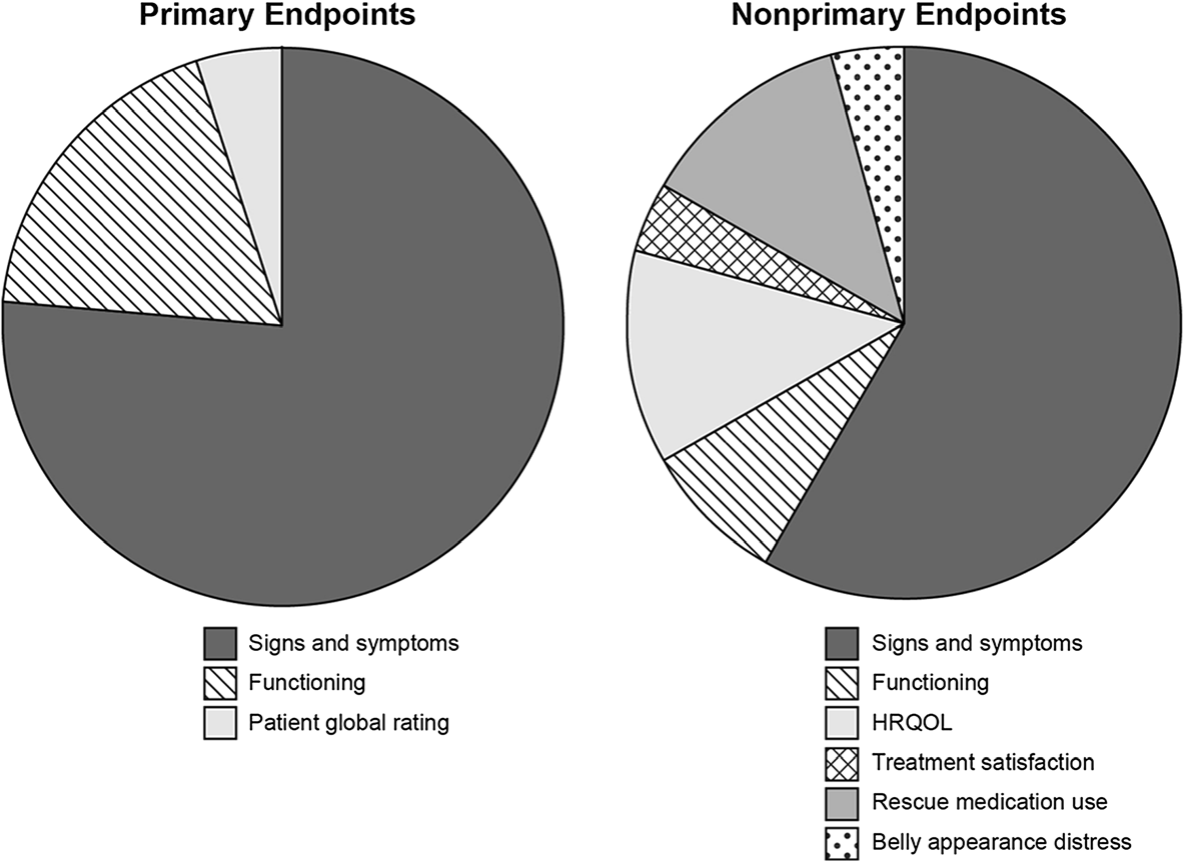
PRO claim based on endpoint status (2000-2012).

Additionally, 19 products received a total of 29 PRO labeling claims using a PRO that was a nonprimary endpoint. These claim types included signs and symptoms (n = 19), functioning (n = 2), HRQOL (n = 3), and other (n = 5) (3 for rescue medication use, 1 for treatment satisfaction, and 1 for belly appearance distress). Importantly, of these, the vast majority were claims based on signs or symptoms, as compared with higher order claims, and were supportive of (but not redundant with) the primary endpoint. However, nonprimary endpoints were more likely to represent claims based on concepts other than signs and symptoms as compared with primary PRO endpoints (Figure 1).

Protonix received a labeling claim of “complete relief of daytime and nighttime heartburn and the absence of regurgitation” based on the patient daily diary as well as an indication of “symptomatic relief.” The primary endpoint in the clinical studies was resolution of macroscopic esophageal lesion. Foradil, Nexium, and Frova followed similar patterns, where the nonprimary PRO endpoint provided key support to a clinical, objective endpoint. Foradil, indicated for “maintenance treatment of asthma and in the prevention of bronchospasm in adults and children” is notable, because the labeling claim specifically denotes, “Foradil demonstrated improvement in many secondary efficacy endpoints, including combined and nocturnal asthma symptom scores, fewer nighttime awakenings and fewer nights in which patients used rescue medication.” Emend (aprepitant) was granted a higher order claim of “A higher proportion of patients receiving Emend reported minimal or no impact of nausea and vomiting on daily life” based on the Functional Living Index-Emesis (FLIE), whereas the primary endpoint was “complete response” or no emesis. Soliris and Letairis also received higher order claims, though in these instances they were in support of HRQOL. Table 2 describes the claim granted and PRO used by product for the 19 products with PRO labeling claims based on nonprimary PRO endpoints.

**Table 2:** Nonprimary endpoints used in medical labeling (2000-2012)

**Product**	**Date of approval**	**Indication**	**PRO measures included**
Protonix	February 2, 2000	Short-term treatment in the healing and symptomatic relief of erosive esophagitis	Daily diary: frequency and severity of acid regurgitation, dysphagia, and daytime and nighttime symptoms
Frova	January 18, 2001	Acute treatment of migraine with or without aura in adults	Headache diary with associated symptoms
Foradil	February 16, 2001	Maintenance treatment of asthma and prevention of bronchospasm in adults and children aged 5 years and older	Daily diary: nighttime asthma symptom score, daytime asthma symptom score, number of inhalations of rescue medication
		Acute prevention of exercise-induced bronchospasm	
Nexium	February 20, 2001	Short-term treatment in the healing and symptomatic resolution of diagnostically confirmed erosive esophagitis	Daily symptom diary
		Maintenance of symptom resolution and healing of erosive esophagitis	
		Treatment of heartburn and other symptoms related to GERD	
		Treatment of patients with H. pylori infection and duodenal ulcer disease	
Elidel	December 13, 2001	Short-term and intermittent long-term therapy in the treatment of mild to moderate atopic dermatitis	Unnamed purities assessment
Emend	March 27, 2003	Prevention of acute and delayed nausea and vomiting associated with initial and repeat courses of highly emetogenic cancer chemotherapy	FLIE
Nevanac	August 19, 2005	Treatment of pain and inflammation associated with cataract surgery	Ocular pain scale
Chantix	May 10, 2006	Aid to smoking cessation treatment	QSU-Brief
			MNWS
			SEI
Vyvanse	February 23, 2007	Treatment of ADHD	CPRS
Soliris	March 16, 2007	Treatment of patients with paroxysmal nocturnal hemoglobinuria to reduce hemolysis	FACIT-Fatigue
			EORTC QLQ-C30
Letairis	June 15, 2007	Treatment of pulmonary arterial hypertension (WHO Group I) in patients with WHO class II or III symptoms to improve exercise capacity and delay clinical worsening	SF-36 Health Survey
Durezol	June 23, 2008	Treatment of inflammation and pain associated with ocular surgery	VAS – eye pain/discomfort
			VAS – photophobia
Ampyra	January 22, 2010	Indicated to improve walking in patients with multiple sclerosis	MSWS-12
Egrifta	November 10, 2010	Reduction of excess abdominal fat in HIV-infected patients with lipodystrophy	Distress associated with belly appearance
Arcapta	July 1, 2011	Long-term, once-daily maintenance bronchodilator treatment of airflow obstruction in patients with COPD, including chronic bronchitis and/or emphysema	Rescue medication use
			Symptom diary
			SGRQ
Firazyr	August 25, 2011	Treatment of acute attacks of hereditary angioedema	Symptom VAS
			Rescue medication
Jakafi	November 16, 2011	Treatment of patients with intermediate or high-risk myelofibrosis, including primary myelofibrosis, postpolycythemia vera myelofibrosis and postessential thrombocythemia myelofibrosis	MFSAF diary
Kalydeco	January 31, 2012	Treatment of cystic fibrosis in patients aged 6 years and older who have a G551D mutation in the CFTR gene	CFQ-R (symptoms domain)
Asclera	March 30, 2010	Treatment of uncomplicated spider veins and uncomplicated reticular veins in the lower extremity	Patient satisfaction using a verbal rating scale

*ADHD*, attention deficit hyperactivity disorder; *CFQ-R*, Cystic fibrosis questionnaire-revised; *CFTR*, cystic fibrosis transmembrane conductance regulator; *COPD*, chronic obstructive pulmonary disease; *CPRS*, Conners’ parent rating scale; *EORTC QLQ-C30*, European organisation for research and treatment of cancer quality of life questionnaire - core questionnaire; *FACIT*, Functional assessment of chronic illness therapy; *FLIE*, Functional living index-emesis; *GERD*, gastroesophageal reflux disease; *MFSAF*, Myelofibrosis Symptom assessment form; *MNWS*, Minnesota nicotine withdrawal scale; *MSWS-12*, 12-item multiple sclerosis walking scale; *PRO*, patient-reported outcome; *QSU-Brief*, Brief questionnaire of smoking urges; *SEI*, Smoking effects inventory; *SGRQ*, St. George’s respiratory questionnaire; *VAS*, visual analogue scale; *WHO*, World health organization.

## Discussion

In the absence of objective clinical endpoints to support efficacy, PRO measures are often used as primary endpoints. PROs may also offer support to a primary endpoint as key secondary, secondary, or exploratory endpoints, which may be either independent of or closely associated with the primary endpoint. Based on this review, PRO labeling claims are more likely to be granted for PROs specified as primary endpoints in confirmatory clinical trials. A variety of reasons could explain the limited number of claims granted based on nonprimary endpoints. The three most prominent reasons are overlapping concepts, commitment to resource, and logistical complexities.

### Overlapping concepts

Primary and nonprimary endpoints may measure the same or very similar constructs. Clinical trial endpoints are intended to characterize a clinical outcome of interest. In an oncology clinical trial, an objective endpoint is survival and a subjective endpoint is a symptom score. In some instances, endpoints may overlap to the point where no new information is supplied by the nonprimary endpoint. Examples are use of a pain scale and a patient diary that measures the same features of pain, multiple functional scales or an event log and a self-reported questionnaire in studies of female sexual dysfunction [13].

### Commitment to resource

Early stages of product development can be an important time to develop new PRO measures. However, sponsors may not commit resources for PRO measures for nonprimary endpoints during the early stages of product development when the likelihood of changes to target product profile and the rate of attrition are still high. The use of inappropriate instruments and the lack of explanation for the choice of PRO measures in clinical trials have been concerns for many researchers [14-16]. However, attention to appropriate measures, even for nonprimary endpoints, may pay dividends. For example, the recent approval of Myrbetriq for the treatment of overactive bladder shows that investing in nonprimary PRO endpoints can add value. The nonprimary PROs were not included in labeling but were still instrumental in the approval of the product, with an advisory board member noting, “I wouldn’t have voted on the affirmative if it wasn’t that there was a signal in the quality of life measures.” Including generic instruments or instruments that do not measure the intended concept in the most appropriate manner in the late stages of product development is unlikely to result in labeling claims.

### Logistical complexities

Inclusion of PRO endpoints in multinational clinical trials brings unique challenges [17]. Culturally adapted PRO instruments, certificates of translation, data collection devices and training manuals in local languages need to be in place at the start of a study. Companies pressed for time may avoid the logistical complexities related to nonprimary PRO endpoints during the execution of a multiregional study. Such complexities include protocol amendments (changes to inclusion and exclusion criteria for patient characteristics while the study is ongoing) and the need to close study centers in some countries and open new centers in other countries (resulting in the need for translations, which cause time delays).

The limited number of US labeling claims based on nonprimary PRO endpoints may also be due to the fact that companies include PROs as nonprimary endpoints in confirmatory clinical trials to satisfy stakeholders other than the FDA, such as other regulatory bodies, health authorities, and third-party payers. As such, there may not have been any attempt to seek US labeling or meet the requirements in the FDA PRO guidance. For example, The Institute for Quality and Efficiency in Health Care in Germany (IQWIG) seeks data to support patient-related benefits and characterizes this view as a type of efficacy [18]. PRO and HRQOL measures may be used in this manner but must demonstrate that they are valid for this purpose.

### Emerging trends

Another distinction worthy of recognition is a product being approved as first in class versus those entering a crowded market. First-in-class therapeutics do not need to rely on differentiation strategies. Sponsors seeking the fastest path to approval may elect to forego secondary PRO endpoints in registration trials and instead seek them in new indications. However, doing so may ultimately be a disadvantage. Including a PRO for these first-in-class products may satisfy regulatory needs (support the primary or biomarker) but also meet HTA needs and set the standard for future competitor products. Companies may be beginning to include PROs as nonprimary endpoints with the rigor required to secure a labeling claim. For example, in the recent approval of Jakafi, a first-in-class treatment for patients with intermediate or high-risk myelofibrosis, a PRO labeling claim was granted on the basis of evidence regarding symptoms related to myelofibrosis, a secondary endpoint in the confirmatory clinical trials [19].

Importantly, on a regulatory level, there may also be a shift in recognition of the importance of PROs as secondary endpoints. A review of Kalydeco, approved in 2011, is instructive. In the confirmatory clinical trials for Kalydeco, a treatment for patients with cystic fibrosis, the primary endpoint was change in percentage predicted FEV_1_, and one of the secondary endpoints was symptoms associated with cystic fibrosis as measured by the Cystic Fibrosis Questionnaire-Revised (CFQ-R). A PRO label was granted for “improved symptoms” based on the CFQ-R, despite reviewer notation in the advisory board minutes that the measure does not meet the standard for validation and development described in the PRO guidance. The reviewer indicated that it was the compelling nature of the data that supported the inclusion of the PRO endpoints in the labeling.

### Guidance on reporting PRO in RCTs

Efforts to improve reporting in RCTs by CONSORT (Consolidated Standards of Reporting Trials) include the reporting of PRO data for trials, including PROs as primary or secondary measures [20]. Such recommendations may improve not only the reporting of PRO data in RCTs but also the planning and execution of PROs so that the suggested data elements may be reliably reported. Ultimately, these improvements could result in an increase in PRO claims granted.

## Conclusions

This review based on 70 PRO labeling claims granted by the FDA between 2000 and 2012 shows that successful PRO labeling claims are typically based on primary endpoints assessing signs and symptoms (81.4%). Although inclusion of PROs as nonprimary endpoints in clinical trials has its challenges, recent PRO labels granted by the FDA show that they can indeed be candidates for PRO labeling claims as long as they can be supported by evidence. The PRO guidance by the FDA has provided the industry with a blueprint for obtaining PRO labels [3]. It is now left to the industry to accept the challenge and recognize the importance of outcomes that matter to all stakeholders, including patients.

## Abbreviations

BLA: Biologic license application; CFQ-R: Cystic fibrosis questionnaire-revised; DAP: Drug approval package; EMA: European medicines agency; FDA: Food and drug administration; FEV1: Forced expiratory volume in the first second of expiration; FLIE: Functional living index-emesis; HRQOL: Health-related quality of life; HTA: Health technology assessment; IQWIG: Institute for quality and efficiency in health care in Germany; NICE: National institute for health and clinical excellence; NME: New molecular entity; PRO: Patient-reported outcome; QoL-AGHDA: Adult growth hormone deficiency scale; US: United States.

## Competing interests

Novartis Pharmaceuticals provided financial support for this research.

## Authors’ contributions

AG conceived of the study idea, reviewed data files and co-authored all drafts. CD reviewed all data files, created initial paper outline, co-authored all drafts. SL, MC and MM reviewed data files, reviewed and contributed to all drafts. All authors read and approved the final manuscript.

## References

[B1] Acquadro C Berzon R Dubois D Leidy NK Marquis P Revicki D Rothman M PRO Harmonization Group: Incorporating the patient’s perspective into drug development and communication: an ad hoc task force report of the Patient-Reported Outcomes (PRO) Harmonization Group meeting at the Food and Drug Administration, February 16, 2001 Value Health 2003 6 522 531 10.1046/j.1524-4733.2003.65309.x 14627058

[B2] Doward L McKenna S Defining patient reported outcomes Value Health 2004 7 Suppl 1 S4 S8 15367236 10.1111/j.1524-4733.2004.7s102.x

[B3] US Department of Health and Human Services, Food and Drug Administration: Guidance for industry Patient-reported outcome measures: use in medical product development to support labeling claims http://www.fda.gov/downloads/Drugs/GuidanceComplianceRegulatoryInformation/Guidances/UCM193282.pdf

[B4] Mordin M Lewis S Gnanasakthy A Patient-reported outcomes as mentioned in product development guidance Value Health 2010 13 A17 A18

[B5] Gnanasakthy A DeMuro C Mordin M Clark M The role of the patient voice in health technology assessment Value Health 2010 13 A19

[B6] Doward LC Gnanasakthy A Baker MG Patient reported outcomes: looking beyond the label claim Health Qual Life Outcomes 2010 8 89 10.1186/1477-7525-8-89 20727176 PMC2936442

[B7] Baldwin M Spong A Doward L Gnanasakthy A Patient-reported outcomes, patient-reported information from randomized controlled trials to the social web and beyond Patient 2011 4 11 17 10.2165/11585530-000000000-00000 21766890 PMC3580131

[B8] Filipovic I Walker D Forster F Curry AS Quantifying the economic burden of productivity loss in rheumatoid arthritis Rheumatology (Oxford) 2011 50 1083 1090 10.1093/rheumatology/keq399 21245074

[B9] Bruner D Movsas B Basch E Capturing the patient perspective: patient-reported outcomes as clinical trial endpointshttp://meetinglibrary.asco.org/content/80-114 10.14694/EdBook_AM.2012.32.80 24451723

[B10] Gnanasakthy A Mordin M Clark M DeMuro C Fehnel S Copley-Merriman C A review of patient-reported outcome labels in the United States: 2006 to 2010 Value Health 2012 15 437 442 10.1016/j.jval.2011.11.032 22583453

[B11] Willke RJ Burke LB Erickson P Measuring treatment impact: a review of patient-reported outcomes and other efficacy endpoints in approved product labels Control Clin Trials 2004 25 535 552 10.1016/j.cct.2004.09.003 15588741

[B12] Food and Drug Administration: Drugs@FDA Drugs@FDAhttp://www.accessdata.fda.gov

[B13] Althoff S Rosen RC DeRogatis L Corty E Quirk F Symonds T Outcome measurement in female sexual dysfunction clinical trials: review and recommendations J Sex Marital Ther 2005 31 153 166 10.1080/00926230590909989 15859374

[B14] Guyatt GH Vanzanten SJOV Feeny DH Patrick DL Measuring Quality of Life in Clinical-Trials - A Taxonomy and Review CMAJ 1989 140 12 1441 1448 2655856 PMC1269981

[B15] Veldhuyzen Van Zanten SJ Quality-of-Life as outcome measures in randomized clinical-trials - An overview of 3 general medical journals Control Clin Trials 1991 12 4 Suppl S234 S242 10.1016/s0197-2456(05)80027-3 1663859

[B16] Lee CW Chi KN The standard of reporting of health-related quality of life in clinical cancer trials J Clin Epidemiol 2000 53 5 451 458 10.1016/S0895-4356(99)00221-8 10812316

[B17] Gnanasakthy A Demuro C Boulton C Integration of patient-reported outcomes in multiregional confirmatory clinical trials Contemp Clin Trials Feb 13;35(1):62–69. [http://www.sciencedirect.com/science/article/pii/S1551714413000141] 10.1016/j.cct.2013.02.006 PMC7105971 23415630

[B18] Johnson R Presentation at the 12th Annual European Congress of the International Society for Pharmacoeconomics and Outcomes Research http://www.ispor.org/congresses/Paris1009/documents/IP8%20-%20Olaf%20Pirk.pdf 10.1111/j.1524-4733.2009.00592_1.x 19796258

[B19] Verstovsek S Mesa RA Gotlib J Levy RS Gupta V DiPersio JF Catalano JV Deininger M Miller C Silver RT Talpaz M Winton EF Harvey JH Jr Arcasoy MO Hexner E Lyons RM Paquette R Raza A Vaddi K Erickson-Viitanen S Koumenis IL Sun W Sandor V Kantarjian HM A double-blind, placebo-controlled trial of ruxolitinib for myelofibrosis New Engl J Med 2012 366 799 807 10.1056/NEJMoa1110557 22375971 PMC4822164

[B20] Calvert M Blazeby J Altman D Revicki D Moher D Brundage M Reporting of patient reported outcomes in randomized trials JAMA 2013 309 8 814 822 10.1001/jama.2013.879 23443445

